# Family and Friends’ Support to People With Dementia in Accessing Older Americans Act Services

**DOI:** 10.1093/geroni/igaf122.2919

**Published:** 2025-12-31

**Authors:** Emerson McSparran, Heather Menne, Kate Singer, Ian Matthew Nelson

**Affiliations:** Miami University, Oxford, Ohio, United States; Miami University, Oxford, Ohio, United States; Miami University, Oxford, Ohio, United States; Miami University, Oxford, Ohio, United States

## Abstract

Some people living with dementia (PLWD) depend on Older Americans Act (OAA) services, including case management, congregate meals, home-delivered meals, homemaker services, and transportation services, to maintain their quality of life and ability to remain independent. Having someone facilitate their access to these services may increase their ability to gain the associated benefits. Using data from the 2023 National Survey of Older Americans Act Participants (NSOAAP), we explored how family and friends are involved in the coordination and use of OAA services for PLWD compared to people without dementia. Descriptive statistics and Fisher’s exact tests were conducted to compare the rates at which participants with dementia and participants without dementia rely on family or friends to help arrange their OAA services. Among PLWD, the proportion of family and friends arranging services was significantly higher than those living without dementia (p<.01). Across services, the proportion of friends and family helping PLWD to arrange services were 56.7% for case management, 34.9% for congregate meals, 65.1% for home-delivered meals, 67.6% for homemaker services, and 48.1% for transportation services. Service providers and policymakers should be aware that more PLWD rely on others to help them access OAA services and account for this in service delivery considerations. While PLWD are not the majority of participants, their perspective is important to the OAA mission of helping older adults live independently.

